# Array Study of VLF Thin-Film Magnetoelectric Antenna with a Microbridge Structure

**DOI:** 10.3390/mi15010011

**Published:** 2023-12-20

**Authors:** Jianhua Jin, Long Jing, Chao Zuo, Zhiling Ma, Yanfeng Shi, Xiaofei Yang, Shi Chen

**Affiliations:** 1 School of Integrated Circuits, Huazhong University of Science and Technology, Wuhan 430074, China; m202172930@hust.edu.cn (J.J.); m202072583@hust.edu.cn (L.J.); d202180887@hust.edu.cn (Z.M.); m202371483@hust.edu.cn (Y.S.); yangxiaofei@mail.hust.edu.cn (X.Y.); 2 Wuhan Second Ship Design and Research Institute, Wuhan 430064, China; cssczc@foxmail.com; 3 Hubei Key Laboratory of Marine Electromagnetic Detection and Control, Wuhan 430064, China

**Keywords:** thin-film magnetoelectric antenna, microbridge structure, array, transmission, reception

## Abstract

Recently, magnetoelectric (ME) antennas have become a hot topic in the field of antenna miniaturization in the very-low-frequency (VLF) band because their size can be reduced to one-ten-thousandth of the size of conventional electric antennas. However, they still suffer from narrow transmission/reception bandwidth and weak radiation intensity. To address these issues, VLF thin-film ME antennas with a microbridge structure are designed, and the method of array connection is used. Test results show that the detection limit of the ME antenna unit is 636 pT/√Hz at 23 kHz and the radiant magnetic field intensity at 0.12 m is 0.87 nT (input power of 10 mW). By series-connecting three ME antenna units with the same resonance frequency, the output response has been increased to 1.72 times and the EM wave radiation intensity is increased to 1.9 times compared to a single unit. By parallel-connecting two ME antenna units with different resonance frequencies, the output response bandwidth has been expanded to 1.56 times compared to a single unit, and the signal radiation bandwidth has been expanded to 1.47 times. This work provides a valuable reference for the future larger-scale arraying of ME antennas.

## 1. Introduction

Very-low-frequency (VLF) electromagnetic (EM) waves are widely used in underwater communications due to their long wavelengths and strong penetration ability [1]. However, the physical size of typical conventional electric antennas needs to be larger than one-tenth of the wavelength corresponding to the operating frequency. As a result, the size of traditional antennas applied in the VLF band can reach 1 to 10 km. The excessively large size can result in high manufacturing costs and integration difficulty. Therefore, the miniaturization of antennas is an urgent problem that needs to be solved.

In recent years, magnetoelectric (ME) antennas based on ferromagnetic/piezoelectric heterostructures have attracted widespread attention as an alternative solution for antenna miniaturization in the VLF band [2,3,4,5]. These antennas leverage the ME coupling effect to convert the mechanical resonance (acoustic waves) excited on the piezoelectric layer into magnetic oscillations in the magnetostrictive layer, subsequently emitting EM waves. They exhibit high radiation efficiency, and the frequency of the radiated EM waves is influenced by the frequency of acoustic waves. At the same frequency, the wavelength of acoustic waves is orders of magnitude smaller than that of EM waves. This allows them to have significantly smaller dimensions compared to traditional antennas. ME antennas can be used as receiving antennas, enabling bidirectional communication, and they can also store the harvested EM wave energy in power devices autonomously. Based on these characteristics, ME antennas have become a hot topic in antenna miniaturization research.

Since the theory of ME antennas was proposed by Yao et al. [2] in 2015, numerous studies have been conducted in this field, leading to substantial progress [3,4,5,6,7,8,9,10,11,12,13]. Currently, a single ME antenna operating in the VLF band has achieved communication distances of up to 120 m [9]. However, as mentioned in our previous work [12], ME antennas suffer from relatively weak output responses and have limited EM wave radiation capabilities, and they exhibit significant ME responses only near resonance frequencies. These limitations hamper their ability to achieve high output responses over a wide bandwidth. Numerous studies have been conducted on broadening the bandwidth and enhancing the output response of ME antennas. Yun et al. [14] designed a composite bulk acoustic resonator based on an Mo/AlN/FeGa thin-film stack with different resonant regions to achieve bandwidth enhancement. The work provides an effective method for broadening bandwidth but does not directly enhance the output response within the operating bandwidth of the ME antenna. Zhuang et al. [15] optimized the magnetomechanical power efficiency of metallic glasses through an opportune annealing condition, thereby enhancing the emission intensity of the ME antenna. This is a promising method for improving the performance of ME devices. However, further research is needed. Dong et al. [16] fabricated ME antennas with high-quality factors based on PZT-8/Metglas and achieved good radiation performance by antenna arrays. Yan et al. [17] effectively broadened the bandwidth of the ME antennas constructed with a sandwich stack of Metglas/PMN-PT/Metglas by parallelly connecting multiple units. These works based on bulk materials have achieved excellent results; however, ME antennas based on bulk materials are not suitable for integration into compact devices.

Compared to bulk ME antennas, thin-film ME antennas are smaller in size, exhibit strong process repeatability, and offer consistent performance across individual antenna units, making them more suitable for large-scale integration. However, traditional thin-film ME antennas with conventional structures (such as cantilever-based ones) pose challenges in terms of fabrication difficulty and vulnerability to mechanical impact, rendering them unsuitable for creating multi-unit ME antenna arrays.

In this article, we have designed a micro-bridge-structure thin-film ME antenna that addresses these issues. This new design offers the advantages of easy fabrication, excellent stability, and integration suitability. Moreover, we have employed an array configuration to enhance its output response and expand its bandwidth.

## 2. Structure and Fabrication

### 2.1. Materials

The choice of piezoelectric and magnetostrictive materials is a crucial factor influencing the performance of ME antennas. Common piezoelectric materials and their properties are shown in Table 1. Polyvinylidene fluoride (PVDF), as an organic piezoelectric material, finds extensive use in the field of flexible wearable devices due to its low density and high flexibility. However, its application scope is limited due to its relatively low piezoelectric coefficient [18]. Lead magnesium niobate-lead titanate (PMN-PT) has the highest piezoelectric coefficient and electromechanical coupling coefficient in the table, but its low Curie temperature and high processing difficulty make it challenging to use in thin-film devices. Lead zirconate titanate (PZT) boasts a high piezoelectric coefficient and excellent dielectric properties, making it widely applied in areas like filters, sensors, and transducers [19]. However, its lead content and inherent material losses limit its further use. In contrast, aluminum nitride (AlN) is lead-free, exhibits good temperature stability, and can be easily fabricated using magnetron sputtering [20]. While the piezoelectric coefficient of AlN is lower compared to piezoelectric ceramic materials, its small dielectric constant, high sound velocity, and compatibility with microfabrication processes make it highly suitable for applications in ME antennas and integrated circuits. Moreover, the low dissipation factor and small dielectric constant of AlN can provide a better signal-to-noise ratio for the receiver application of frequencies over 1 kHz [21]. Considering these factors, AlN is chosen as the material of the piezoelectric layer for the ME antenna.

Correspondingly, the properties of common magnetostrictive materials are shown in Table 2. NiFe_2_O_4_, being a ferrite, has a relatively small magnetostriction coefficient, making it unsuitable for use in ME antenna applications. Terfenol-D is a giant magnetostrictive material, with the highest magnetostriction coefficient in the table, at 1400 ppm. However, it has a lower magnetic permeability, requiring a significant DC bias magnetic field to reach saturation. Additionally, Terfenol-D is prone to oxidation and is challenging to integrate into compact devices. In contrast, Metglas2605 offers high magnetic permeability and requires only a small DC bias magnetic field to reach saturation. However, its magnetostriction coefficient is relatively small, resulting in a lower output response. FeCoSiB shares the characteristics of high magnetic permeability, high piezomagnetic coefficient, and a substantial magnetostriction coefficient. Moreover, it has a low resistivity, making it suitable as a conductive layer in devices. Considering these factors, FeCoSiB is chosen as the material of the magnetostrictive layer for the ME antenna.

**Figure 1:**
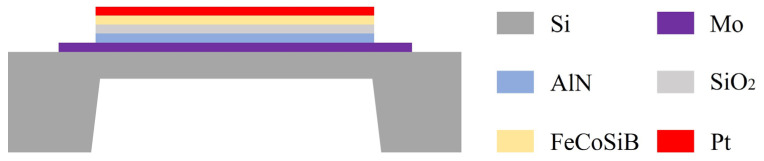
Diagram of microbridge ME antenna.

A thin-film ME antenna typically consists of a multi-layered structure comprising a magnetostrictive layer, a piezoelectric layer, a bottom electrode, and a substrate. Silicon is commonly used as the substrate for compatibility with microelectronics processes, facilitating the future integrated production of ME antennas. However, the presence of the substrate affects the antenna’s performance. When the device vibrates, the silicon substrate can clamp the functional layer, thereby weakening the device’s output [23]. Additionally, the device’s resonance frequency increases with an increase in substrate thickness, limiting its low-frequency response. To address this issue, it is necessary to etch the silicon substrate at the bottom to reduce clamping and enhance the device’s performance. Considering factors such as fabrication complexity, response output, and stability, a micro-bridge ME structure has been designed, as shown in Figure 1. This bridge-like structure fixes the functional layer at both ends, with the silicon layer left at the bottom serving as a supporting layer to enhance the device’s vibration resistance. By controlling dimensional parameters such as functional layer length, thickness, depth of silicon substrate etching, and volume of silicon substrate etching, ME antennas with different resonance frequencies can be obtained for various applications. Compared to cantilever-based structures, the microbridge structure is simpler, more stable, and has lower fabrication complexity, making it suitable for large-scale integration.

### 2.2. Simulation

**Figure 2:**
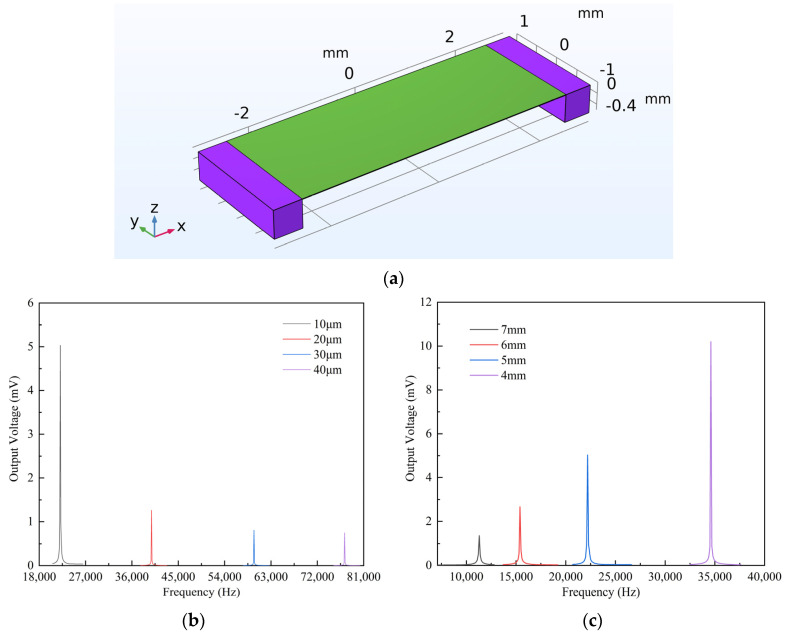
(**a**) Simulation model of microbridge ME antenna; (**b**) Relationship between the response and substrate thicknesses; (**c**) Relationship between the response and functional layer length.

In order to achieve a better performance, the structure of the ME antenna is optimized through COMSOL with the simulation model of the microbridge ME antenna shown in Figure 2a. A 3D model is used, and the electrostatics module, magnetic field module, and solid mechanics module are added. The thin SiO_2_ layer and the bottom electrode are ignored in the model, and the bottom of both ends are set as fixed. The three-layer structure of Si/AlN/FeCoSiB is set as a linear elastic material, a piezoelectric material, and a magnetostrictive material, respectively, with the corresponding physical fields added. Considering the computational accuracy and solution time, the air domain is solved with a regular mesh, and the piezoelectric, magnetostrictive, and solid mechanics domains are solved with a refined mesh. In this work, the steady-state solver is chosen to simulate the characteristics of the ME antenna in the steady state, and the small-signal solver is chosen to simulate the characteristics of the magnetoelectric antenna in the frequency domain, so as to obtain the resonant frequency and output response of the device.

ME antennas have two vibration modes: bending vibration mode and longitudinal vibration mode. The bending vibration mode has a smaller response, but its resonance frequency is much lower, making it suitable for low-frequency applications. Because the double ends of the microbridge structure are fixed, the designed device works as a clamped–clamped beam that operates in the bending vibration mode. According to [24], the natural frequency of the device with *N* layers can be expressed as:

(1) $$\omega_{i}=\frac{\lambda_{i}^{2}}{l^{2}}\sqrt{\frac{D}{\mu }}$$

where $\lambda_{i}$ is a constant related to the vibration mode, l is the length of the beam, *D* is the equivalent flexural rigidity, and μ is the mass per unit area. *D* can be expressed as:

(2) $$D=\int_{0}^{t_{total}}Y_{11,n}^{\prime }z^{2}dz$$

where $t_{total}$ is the total thickness of the laminate and $Y_{11,n}^{\prime }$ is the plate modulus of the *n*-th layer. μ can be expressed as:

(3) $$\mu \;=\sum_{n=1}^{N}t_{n}\rho_{n}$$

where $t_{n}$ and $\rho_{n}$ denote the thickness and mass density of the *n*-th layer, respectively.

It is evident that the thickness of the silicon substrate affects the equivalent flexural rigidity and the mass per unit area and thus the resonance frequency. Meanwhile, when the functional layer vibrates under the influence of a magnetic field, the bottom silicon substrate also vibrates and clamps the functional layer. Therefore, the thickness of the silicon substrate will affect the ME response.

In order to study the optimal substrate thickness, the functional layer’s length is initially set to 5 mm, and the substrate thicknesses are set to 10 μm, 20 μm, 30 μm, and 40 μm. Simulation results are shown in Figure 2b. It can be observed that the resonance frequencies for the four substrate thicknesses are 22.2 kHz, 39.8 kHz, 58.7 kHz, and 77.3 kHz, respectively, with corresponding output voltages at resonance frequencies of 5.03 mV, 1.26 mV, 0.81 mV, and 0.75 mV. A thicker substrate leads to a higher resonance frequency and a smaller ME response at resonance. Therefore, whether to reduce the resonance frequency or increase the ME response, a reduction in substrate thickness is necessary. However, an excessively thin substrate does not provide sufficient support for the functional layer, affecting the device’s stability. Considering all factors, a substrate thickness of 10 μm is chosen as it can provide a significant ME response while maintaining device stability.

The length of the functional layer also affects the ME response. Setting the bottom silicon thickness of the functional layer to 10 μm and the resonance frequency to 30 kHz, the calculated functional layer length is approximately 5 mm. Therefore, we set four functional layer lengths to 4 mm, 5 mm, 6 mm, and 7 mm, respectively. Simulation results are shown in Figure 2c. The resonance frequencies for the four functional layer lengths are 34.6 kHz, 22.2 kHz, 15.41 kHz, and 11.32 kHz, respectively, with corresponding output voltages at resonance frequencies of 10.27 mV, 5.03 mV, 2.66 mV, and 1.35 mV. A longer functional layer results in a lower resonance frequency, consistent with theoretical calculations. In the process of designing a VLF ME antenna, considering both the resonance frequency and the magnitude of the ME response, a functional layer length of 5 mm is chosen, allowing the antenna to maintain a large ME response while operating within the VLF band.

**Figure 3:**
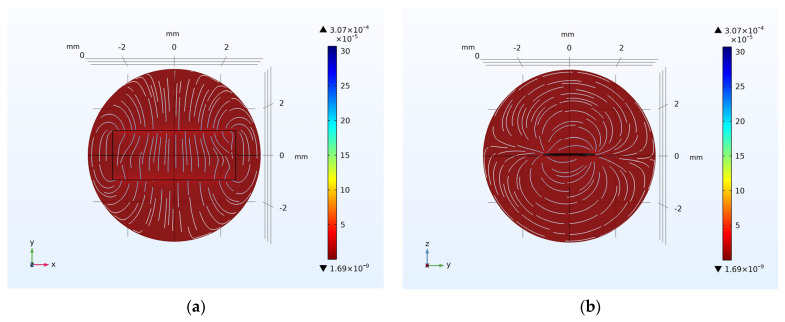
Near-field radiation patterns of the ME antenna: (**a**) XOY plane; (**b**) YOZ plane.

The simulation of the transmitting performance has also been conducted. Figure 3a,b shows the radiation patterns of $H_{\theta }$ and $H_{r}$, respectively. It is evident that in the near-field region of the antenna, the radiation pattern of the $H_{\theta }$ component is “∞”-shaped and the radiation pattern of the $H_{r}$ component is “8”-shaped. It can be observed that the ME antenna exhibits significant directionality in its radiation.

### 2.3. Fabrication

To ensure process consistency, eight ME antenna units are fabricated on the same chip. The chip chosen is a customized AlN substrate from SITRI (Shanghai Industrial μTechnology Research Institute, Shanghai, China), which consists of a three-layer structure: 500 μm thick Si, 50 nm thick Mo, and 1 μm thick AlN (002). Subsequent processes are conducted on this chip. The AlN film is structured by inductively coupled plasma (ICP) etching, while the Mo layer is structured by reactive ion etching (RIE) to ensure isolation between multiple ME antenna units. The Mo layer serves as bottom electrode. A 10 nm thick SiO_2_ film is deposited on the AlN using a plasma-enhanced chemical vapor deposition (PECVD) process at 100 °C, to protect the AlN from being etched by the developer solution in subsequent processes [25,26]. A 1 μm thick FeCoSiB layer is deposited on the SiO_2_ by magnetron sputtering, with a 40 nm thick Pt layer above as a top electrode for protection against oxidation. Subsequently, annealing is conducted at 350 °C under a magnetic field of 1000 Oe to induce the easy axis direction.

**Figure 4:**
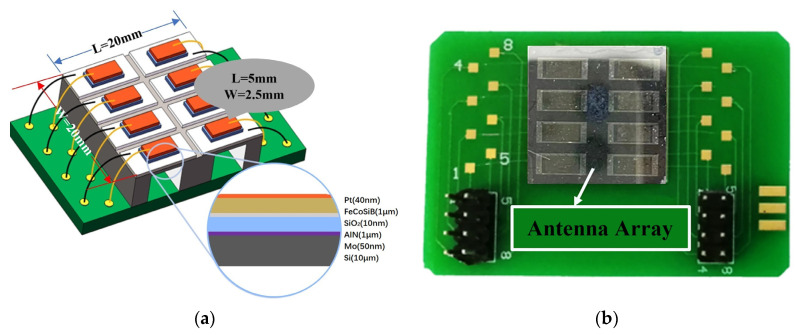
Microbridge-type ME antennas: (**a**) Diagram of the designed device; (**b**) Photograph of the designed device.

To increase the output response and reduce the resonance frequency of the ME antenna, it is necessary to etch the bottom silicon substrate. A deep silicon etching process is employed to etch the backside of the silicon substrate to a depth of 490 μm, resulting in a silicon substrate thickness below the functional layer of 10 μm. Different silicon substrate mask sizes are used to achieve ME antenna units with different resonance frequencies. Finally, the Mo and Pt electrodes of the antennas are connected to the pads on a PCB using the wire bonding process. The fabricated microbridge-type ME antennas are shown in Figure 4, with each unit having functional layer dimensions of 5 mm × 2.5 mm.

## 3. Experiments and Analysis

### 3.1. Performance of Single ME Antenna

**Figure 5:**
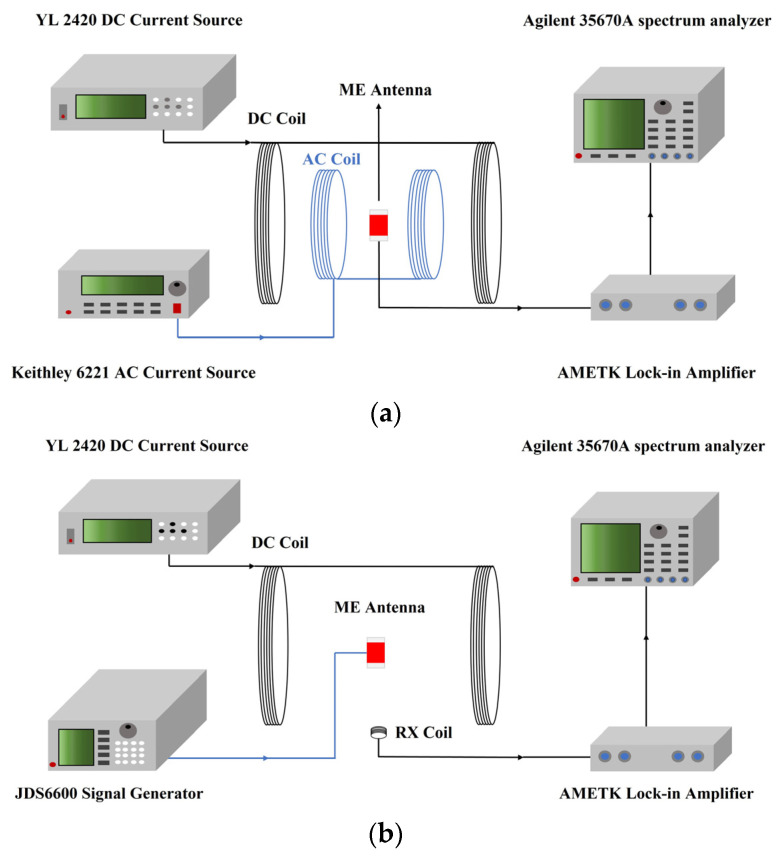
(**a**) ME antenna receiving test system; (**b**) ME antenna transmitting test system.

Referring to the work in [12], the receiving and transmitting performance of a microbridge ME antenna unit were first tested. The test systems are shown in Figure 5.

**Figure 6:**
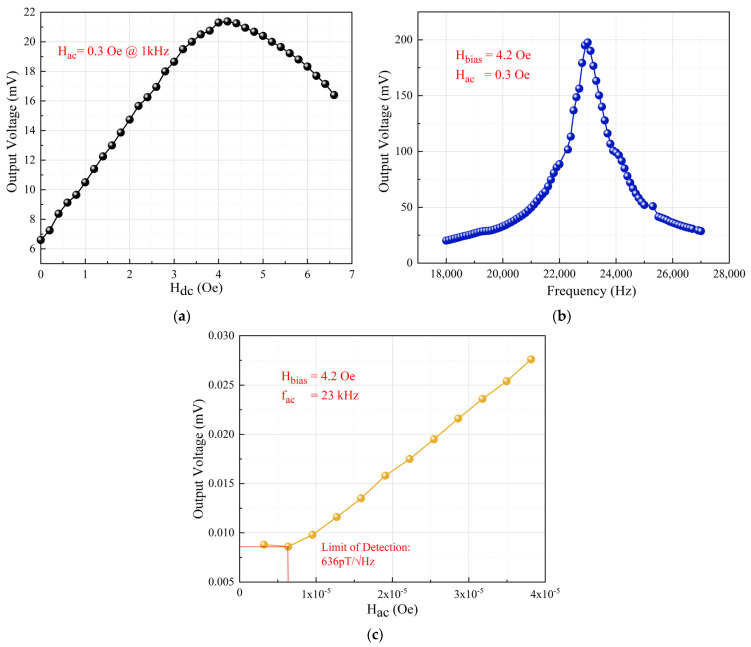
Receiving performance: (**a**) Relationship between the ME output voltage and the intensity of the DC bias magnetic field; (**b**) The frequency response curve; (**c**) The detection limit.

Figure 6a shows the ME response voltage of the ME antenna unit as a function of the DC bias magnetic field under a 1 kHz AC magnetic field with an amplitude of 0.3 Oe. From Figure 6a, it can be observed that the output response of the ME antenna unit initially increases linearly with the magnitude of the DC magnetic field. As the magnetic field approaches saturation, the growth trend of output response slows down and reaches its maximum at a magnetic field magnitude of 4.2 Oe. Therefore, the optimal DC bias for the ME antenna unit is 4.2 Oe.

Figure 6b shows the frequency dependence of the output response, measured from the ME antenna unit under an AC magnetic field of 0.3 Oe, and the optimal DC bias field. As the frequency of the AC magnetic field increases, the ME antenna unit’s output response gradually increases. When the frequency of the AC magnetic field matches the resonance frequency of the ME antenna unit, the output response reaches its maximum value. Continuing to increase the frequency of the AC magnetic field will cause the output response to rapidly decrease. This is a result of the resonance enhancement characteristic of ME composite materials, where the ME output response at the resonance frequency is several times or even tens of times greater than that at non-resonance frequencies. The test results indicate that the resonance frequency of the ME antenna unit is 23 kHz.

Figure 6c displays the detection limit test results at the resonance frequency. The detection limit test was conducted by setting an optimal DC bias magnetic field of 4.2 Oe and fixing the frequency of the AC magnetic field at 23 kHz while gradually reducing the amplitude of the AC magnetic field. The test results revealed that the detection limit of the ME antenna unit is approximately 636 pT/√Hz.

**Figure 7:**
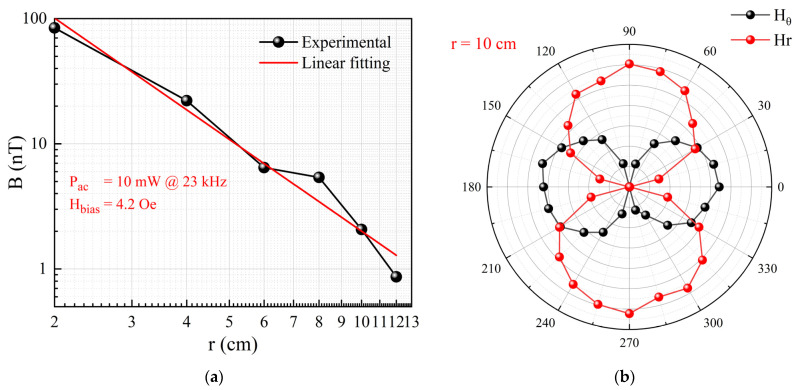
Transmitting performance: (**a**) Relationship between the radiation magnetic field intensity and distance; (**b**) XOY plane radiation pattern.

During the transmitting performance testing of the ME antenna unit, an optimal DC bias magnetic field of 4.2 Oe was also applied. An AC signal with a power of 10 mW was applied to the piezoelectric layer using a JDS6600 signal generator (Hangzhou Junce Instrument, Hangzhou, China), causing the ME antenna unit to radiate EM waves. By changing the relative position between the receiving coil and the ME antenna unit, a relationship curve between the radiation intensity and the radiation distance was obtained, as shown in Figure 7a. Through linear fitting, the slope of this curve was determined to be −2.435, indicating that the radiation intensity decreases linearly with *r*^−2.435^. This result shows a certain deviation from the near-field radiation of a magnetic dipole, which exhibits linear decay as *r*^−3^. Possible reasons for this deviation are twofold. Firstly, the piezoelectric layer itself possesses a certain EM wave radiation capability, and the radiation can be equivalently modeled as an electric dipole radiation. In the near-field region, the radiation intensity exhibits linear decay as *r*^−2^, as mentioned in [12]. Secondly, due to the relatively short distance of radiation, there may be some measurement errors. Experimental results confirm the potential of the ME antenna to serve as a transmitting antenna.

Using the center of the ME antenna unit as the origin (XOY plane), measurements of magnetic field intensity were taken at various angles on a circle with a radius of *r* = 10 cm. This provided the radiation pattern of the ME antenna unit’s *H_r_* and *H_θ_* components in the near-field region, as shown in Figure 7b. For the *H_r_* component, the maximum radiation intensity occurred at 90° and 270° and was about 2.6 nT, and the minimum radiation intensity occurred at 0° and 180° and was about 0. Conversely, for the *H_θ_* component, the maximum radiation intensity occurred at 0° and 180° and was about 1.2 nT, and the minimum radiation intensity occurred at 90° and 270° and was about 0. These test results align with the radiation pattern of a magnetic dipole and our simulation, demonstrating clear directionality.

### 3.2. Performance of Series Connection

**Figure 8:**
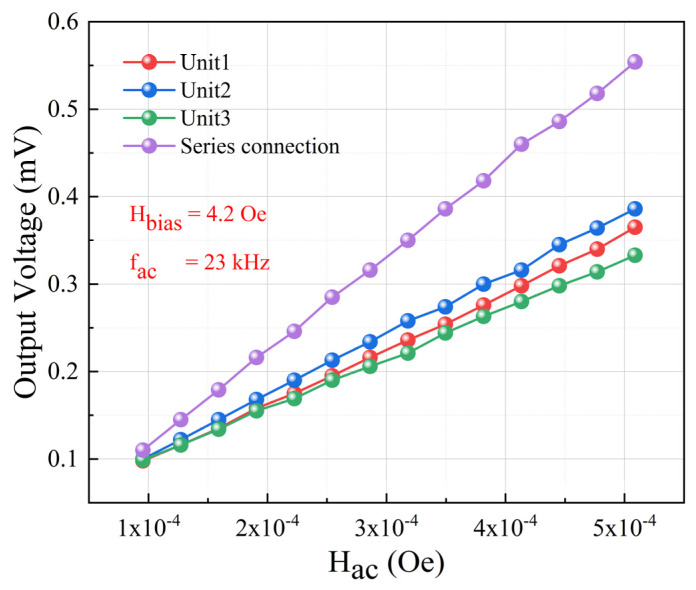
Receiving performance: test results for the ME output voltage before and after series connection.

In order to study the effect of series connection on the receiving performance of ME antennas, three units with similar resonance frequencies and ME coupling coefficients were selected for testing and analysis. The test results are shown in Figure 8. A DC bias magnetic field of 4.2 Oe was set, and the AC magnetic field frequency was 23 kHz. The slope of the curves represents the sensitivity of the antennas, with sensitivity values of 132 V/T, 122.6 V/T, and 113.2 V/T for the three individual units. These results demonstrate that the fabricated thin-film ME antennas exhibit better consistency compared to our previous work [12] with bulk material. When the three units were connected in series, a significant enhancement in the ME output response was achieved, with a sensitivity of 211 V/T, approximately 1.72 times that of a single ME antenna unit. According to the series-parallel equivalent circuit model [27], the device’s ME output response is related to the capacitance before and after series connection. Therefore, a digital multimeter was used to measure the capacitance of the three individual ME antenna units and the total capacitance after serial connection. The capacitance values before series connection were 8.456 nF, 8.247 nF, and 8.024 nF for the three units, while the total capacitance after series connection was 4.581 nF, as shown in Table 3. The capacitance of an individual unit is approximately 1.8 times that of the total capacitance after being connected in series, which is very close to the factor of 1.72 by which the sensitivity of the ME antenna units changes before and after being connected in series. According to the theoretical model [27], the sensitivity after series connection should be three times that of a single unit. The discrepancy between the test results and theoretical calculations is likely due to interface charge loss. These experimental results demonstrate that connecting multiple ME antenna units in series can significantly enhance the output response and improve the sensitivity. This approach proves to be an effective method for enhancing the performance of thin-film ME antenna devices.

**Figure 9:**
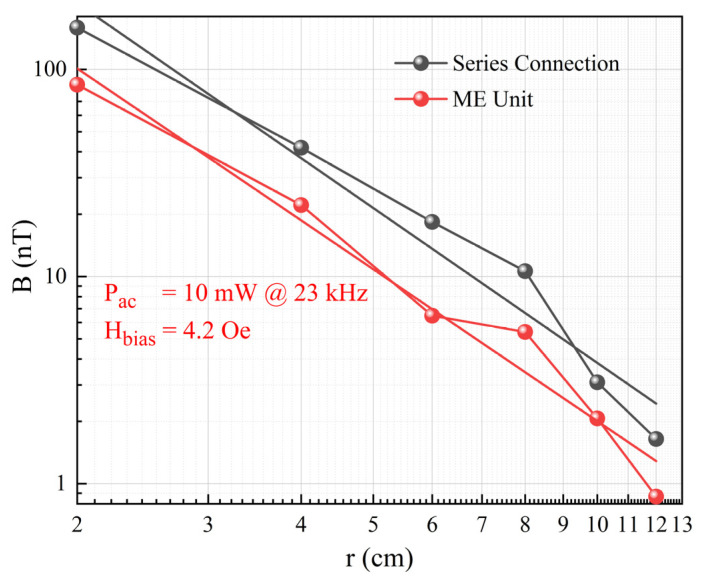
Transmitting performance: test results for radiation magnetic field intensity of ME antennas before and after series connection.

Similarly, we tested the effect of series connection on the transmitting performance. The test results for the radiation intensity and distance of the individual ME antenna unit and the series-connected ME antennas are shown in Figure 9. It can be observed that after series connection, the output response of the ME antenna has been significantly enhanced and is approximately 1.9 times that of the individual ME antenna unit’s radiation intensity. Due to factors such as charge loss, the actual amplification factor is smaller than the theoretical calculation. The experimental results demonstrate that series connection greatly improves the ability of ME antennas to radiate EM waves. This capability can be further enhanced by large-scale series connection to extend the radiation distance.

### 3.3. Performance of Parallel Connection

**Figure 10:**
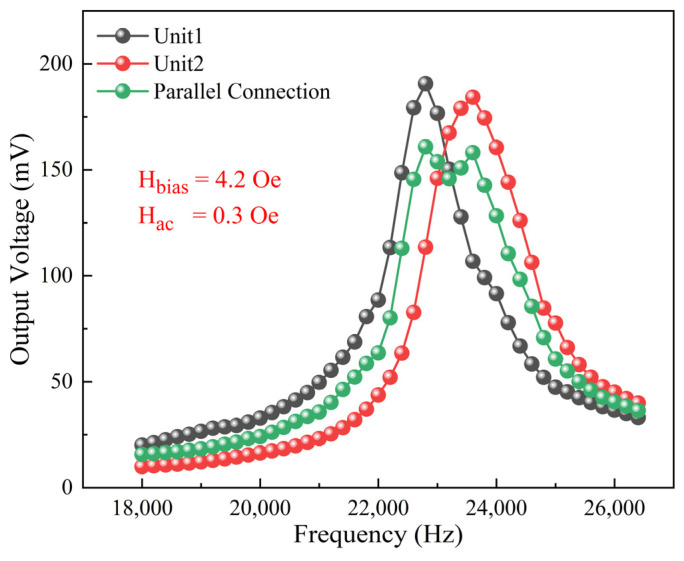
Receiving performance: test results for frequency response of ME antennas before and after parallel connection.

In order to study the effect of parallel connection on the receiving performance of the ME antenna, two units with different resonance frequencies were selected for testing and analysis. During the testing process, the amplitude of the DC bias magnetic field and AC magnetic field were set to 4.2 Oe and 0.3 Oe, respectively. Test results are shown in Figure 10. It can be seen that the resonance frequencies of the two single units were quite different—22.8 kHz and 23.6 kHz, respectively—and the ME output responses at the resonance frequencies were 190.59 mV and 184.24 mV, with 3 dB bandwidths of 1100 Hz and 1600 Hz. The ME output response of the device after the parallel connection was 160.83 mV, with a 3 dB bandwidth of 2100 Hz, which is about 1.56 times that of a single unit. Through the parallel connection of the ME antenna units with different resonance frequencies, although the maximum ME response of the device has decreased to a certain extent, the 3 dB bandwidth has been significantly broadened, which indicates that the ME antennas can maintain a higher ME response in a wider frequency range after parallel connection, thus broadening their frequency application range.

**Figure 11:**
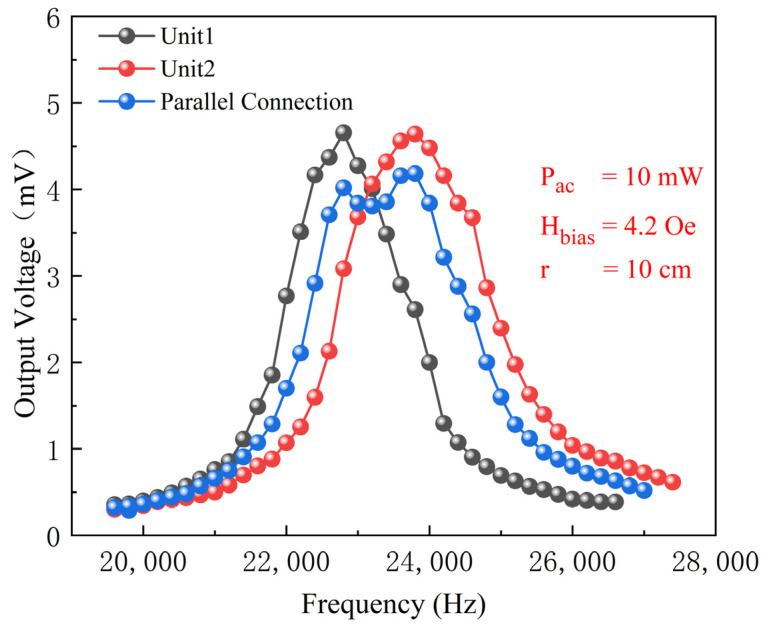
Transmitting performance: relationship between the output voltage of the Rx coil and the frequency of the AC magnetic field before and after parallel connection of ME antennas.

In the study of the effect of parallel connections on the ME antenna’s transmitting performance, two ME antenna units with different resonance frequencies were connected in parallel. A DC bias magnetic field of 4.2 Oe was set, and the output power of the signal generator was 10 mW. The EM wave signal was received using an Rx coil at a distance of 10 cm from the antenna, and test results are shown in Figure 11. Since the capacitance of the two ME antenna units is different, the intensity of the EM wave signals radiated by the paralleled antennas decreases somewhat. The 3 dB bandwidths of the individual ME antenna units are 1400 Hz and 1600 Hz, respectively. After parallel connection, the antenna’s 3 dB bandwidth is 2200 Hz, which is approximately 1.47 times that of the individual ME antenna unit’s 3 dB bandwidth. Therefore, it is possible to expand the antenna’s radiation frequency range by parallel connecting multiple ME antennas.

### 3.4. Comparison with Reported ME Antennas

The research on ME antenna carried out so far in the VLF band is mainly based on bulk materials, and there is very little on thin-film materials. The VLF thin-film ME antenna with a microbridge structure designed in this paper has a significantly smaller size and relatively wider bandwidth compared with other reported VLF ME antennas, as shown in Table 4. Meanwhile, it is compatible with microelectronic process and more suitable for integration into compact devices and large-scale integration.

## 4. Conclusions

Thin-film ME antennas have the characteristics of small size, low power consumption, and easy integration and arraying compared with the traditional electric antenna, which has good application prospects in the fields of undersea communication, biomedical science, weak magnetic detection, and wearable devices. In view of the problems of the large size of VLF antennas, we designed and prepared a thin-film ME antenna based on FeCoSiB/AlN and conducted in-depth research on the receiving and transmitting performance of the ME antenna as well as the effect on the device performance after series-parallel connection, and the main research results are as follows.

(1) A microbridge thin-film ME antenna with FeCoSiB as a magnetostrictive phase and AlN as a piezoelectric phase has been designed with an operating bandwidth in the VLF band and a size of only 5 × 2.5 mm. The ME antenna reaches a detection limit of 636 pT/√Hz with an optimal DC-biased magnetic field of 4.2 Oe and a resonance frequency of 23 kHz. The fabricated thin-film ME antennas exhibit better consistency compared to ME antennas with bulk material. The antenna’s radiation intensity reaches 3.1 nT at a distance of 0.1 m and can be further enhanced by array connections. The fabricated ME antenna exhibits good transmitting and receiving performance.

(2) By series-connecting three ME antenna units of the same resonance frequency on the same substrate, the output response of the device is increased to 1.72 times that of a single unit, while the radiation intensity of the EM wave is increased to 1.9 times, which demonstrates that series connection effectively improves the ME output response and the radiation capability of the ME antenna. By parallel connecting two ME antenna units with different resonance frequencies on the same substrate, the 3 dB bandwidth of the ME output response is broadened to 1.56 times that of the single unit, while the 3 dB bandwidth of the EM wave radiation intensity is broadened to 1.47 times, which demonstrates that parallel connection effectively broadens the operating bandwidth of the ME antenna. This work provides a valuable reference for the future larger-scale arraying of ME antennas.

## Figures and Tables

**Table 1:** Properties of common piezoelectric materials [22].

	BaTiO_3_	PZT	PMN-PT	AlN	PVDF
*d*_31_ (pC/N)	−90	−175	~700	−2	16.5
*d*_33_ (pC/N)	191	400	2000	5.5	−33
*ɛ*_r_	1700	1750	5000	10.5	10
*ρ* (g/cm^3^)	6	7.7	7.8	3.3	1.78
*K*_33_	0.63	0.72	0.9~0.94	0.24	0.19

**Table 2:** Properties of common magnetostrictive materials [22].

	NiFe_2_O_4_	Terfenol-D	Fe-Ga	Metglas2605	FeCoSiB
*d*_33_ (nm/A)	-	25	20	-	50.3
*λ*_S_ (ppm)	27	1400	200	40	158
*μ*_r_	20	6~10	20	>40,000	70,000
*ρ* (g/cm^3^)	5.37	7.8	7.7	7.18	7.59
*K*_33_	-	0.44	-	0.37	-

**Table 3:** Results of capacitance test for ME antenna units and series connection structure.

ME Antenna Unit	Unit 1	Unit 2	Unit 3	Series Connection
Capacitance (nF)	8.456	8.247	8.024	4.581

**Table 4:** Comparison between this work and published work.

Reference	Center Frequency	Radiation Intensity	Relative Bandwidth	Size of a Single Unit	Composition
[7]	28.1 kHz	1 nT @ 1.35 m, 100 mW	-	80 × 20 mm^2^	PZT/Metglas
[12]	26 kHz	1.06 nT @ 1.2 m, 10 mW	3.7%	60 × 20 mm^2^	PZT/Metglas
[16]	23.7 kHz	200 nT @ 1 m, -	0.83%	80 × 9 mm^2^	PZT/Metglas
This work	23 kHz	3.1 nT @ 0.1 m, 10 mW	9.6%	5 × 2.5 mm^2^	AlN/FeCoSiB

## Data Availability

All the necessary data are included in the article.
